# Prognostic significance of sarcopenia and systemic inflammation for patients with renal cell carcinoma following nephrectomy

**DOI:** 10.3389/fonc.2022.1047515

**Published:** 2022-12-15

**Authors:** Qiuchen Liu, Jiajian Yang, Xin Chen, Jiakang Yang, Xiaojun Zhao, Yuhua Huang, Yuxin Lin, Jinxian Pu

**Affiliations:** ^1^ Department of Urology, The First Affiliated Hospital of Soochow University, Suzhou, China; ^2^ Department of Radiology, Changhai Hospital, Second Military Medical University, Shanghai, China; ^3^ Department of Urology, Dushu Lake Hospital Affiliated to Soochow University, Suzhou, China

**Keywords:** nomogram, renal cell carcinoma, sarcopenia, systemic inflammation, prognosis

## Abstract

**Background:**

To clarify the prognostic effect of preoperative sarcopenia and systemic inflammation, and to develop a nomogram for predicting overall survival (OS) of patients with renal cell carcinoma (RCC) following partial or radical nephrectomy.

**Methods:**

Patients with RCC following nephrectomy from the First Affiliated Hospital of Soochow University during January 2018 to September 2020 were included in this study. The relationship between sarcopenia and inflammatory markers was identified by logistic regression analysis. Then univariable Cox regression analysis, LASSO regression analysis and multivariable Cox regression analysis were analyzed sequentially to select the independent prognostic factors. Kaplan-Meier survival curves were applied to ascertain the prognostic value. Finally, the identified independent predictors were incorporated in a nomogram, which was internally validated and compared with other methods.

**Results:**

A total of 276 patients were enrolled, and 96 (34.8%) were diagnosed with sarcopenia, which was significantly associated with neutrophil-to-lymphocyte ratio (NLR). Sarcopenia and elevated inflammation markers, i.e., NLR, platelet-to-lymphocyte ratio (PLR) and the modified Glasgow Prognostic Score (mGPS), were independent factors for determining the OS. The model had good discrimination with Concordance index of 0.907 (95% CI: 0.882–0.931), and the calibration plots performed well. Both net reclassification index (NRI) and integrated discriminant improvement (IDI) exhibited better performance of the nomogram compared with clinical stage-based, sarcopenia-based and integrated “NLR+PLR+mGPS” methods. Moreover, decision curve analysis showed a net benefit of the nomogram at a threshold probability greater than 20%.

**Conclusions:**

Preoperative sarcopenia was significantly associated with NLR. A novel nomogram with well validation was developed for risk stratification, prognosis tracking and personalized therapeutics of RCC patients.

## Introduction

Renal cell carcinoma (RCC) is the most common solid lesion of the kidney, accounting for approximately 85% of all kidney malignancies and 3% of systemic malignancies, with about 76% of 5-year relative survival (1). RCC comprises three main types: clear cell RCC (ccRCC), papillary RCC (pRCC) and chromophobe RCC (chRCC), among which ccRCC has the worst prognosis. Although numerous prognostic indices, e.g., the International Society of Urological Pathology (ISUP) grading system, Tumor Node Metastasis (TNM) staging system and performance status, have been developed, they are limited in applicability, singleness and subjectivity. Considering up to 20-40% postoperative tumor recurrence rate of RCC predicting reduced survival (2), how to use preoperative routine examination for early identification of patients at high risk of adverse treatment outcomes and premature mortality is still a clinical priority. Actually, the International Metastatic Renal Cell Carcinoma Database Consortium (IMDC) score, a well-established prognostic model with a combination of serum inflammatory markers and Karnofsky performance status for risk stratification of metastatic RCC (3), provides new perspectives in clinic.

Accumulating studies confirmed strong associations between sarcopenia and poor prognosis of malignancies, including RCC (4–6). Here sarcopenia is defined as age-related loss of skeletal muscle mass, as well as low muscle strength and physical performance (7). As a hallmark of localized and metastatic tumors, systemic inflammation is also hypothesized to be integral to the progression of sarcopenia and cancer cachexia (8–10). Moreover, the combination of sarcopenia and inflammation could lead to worse prognosis of malignant tumors (11–13). In RCC studies, sarcopenia with elevated inflammation, e.g., the modified Glasgow Prognostic Score (mGPS), neutrophil-to-lymphocyte ratio (NLR) and C-reactive protein (CRP), was investigated for predicting inferior overall survival (OS) (14, 15). Although systemic inflammation was proved to be associated with sarcopenia risk in RCC patients of China (16), the combined impact of these two factors on survival have not been well explored yet.

In this study, the independent and combined impacts of preoperative sarcopenia and systemic inflammatory markers on prognosis of RCC were evaluated, and a novel informatics model based on sarcopenia and inflammatory markers was developed and validated for preoperatively predicting prognosis of RCC patients following partial nephrectomy (PN) or radical nephrectomy (RN) in the era of precision medicine and intelligent healthcare.

## Materials and methods

### Study patients

**Figure 1:**
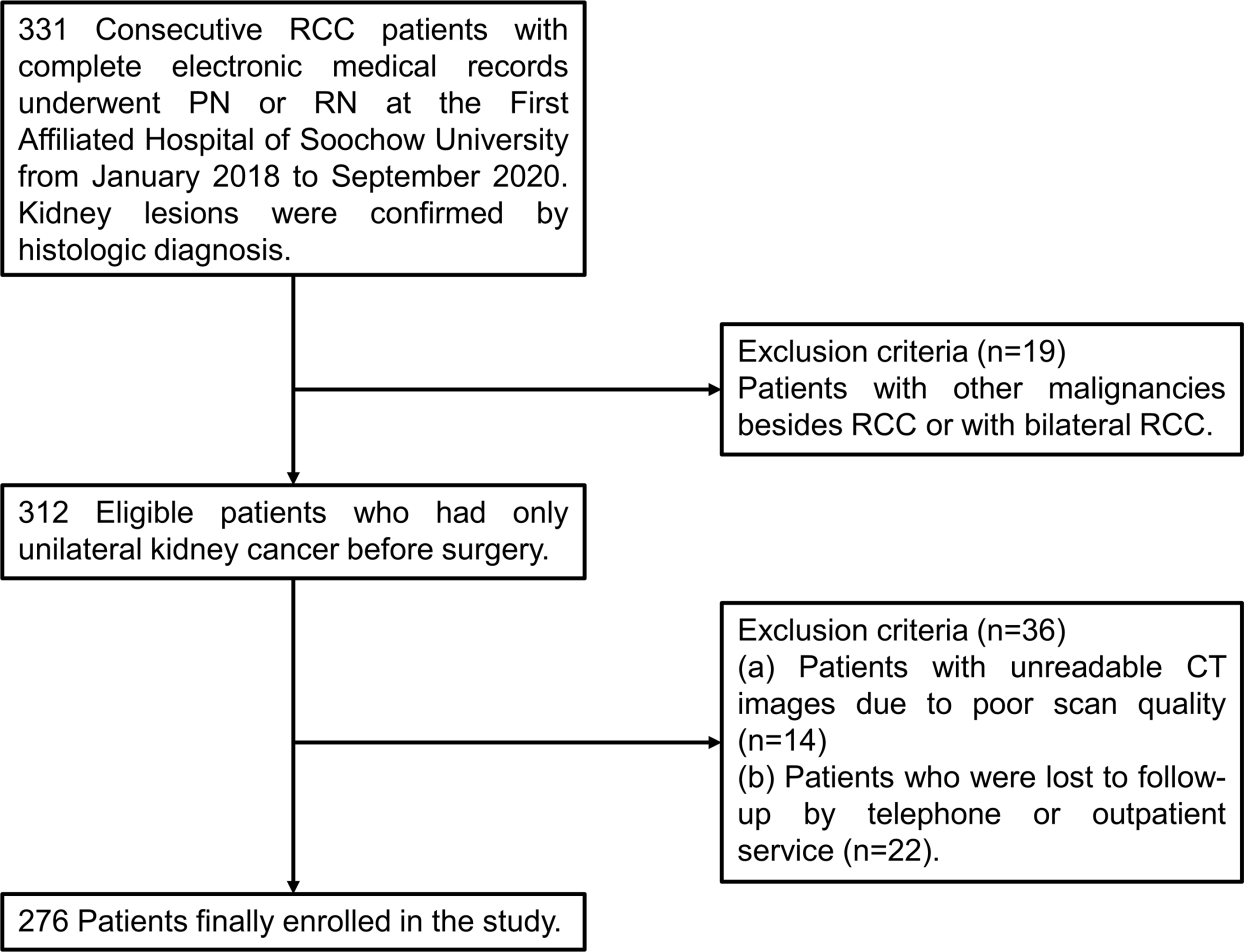
A flow chart of screening of RCC patients. RCC, renal cell carcinoma; PN, partial nephrectomy; RN, radical nephrectomy; CT, computerized tomography.

Data of patients who received a diagnosis of stage I to IV RCC and underwent PN or RN at the First Affiliated Hospital of Soochow University from January 2018 to September 2020 were collected and reviewed retrospectively. The inclusion criteria were set as follows: (I) age of 18 years or older; (II) a confirmed histologic diagnosis of RCC; (III) complete electronic medical records including computerized tomography (CT) images within one month before surgery and clinical laboratory tests within one week before surgery. The exclusion criteria were applied: (I) patients with other malignancies besides RCC or with bilateral RCC (n=19); (II) unreadable CT images due to poor scan quality (n=14); (III) patients who were lost to follow-up by telephone or outpatient service (n=22). As shown in **Figure 1**, a total of 276 patients were finally selected for further statistical analysis. The TNM staging system was performed according to the corresponding eighth edition of the American Joint Committee on Cancer (AJCC) Staging Manual (17).

### Sarcopenia and its measurement

Considering the advantages of CT examinations in body composition quantification (18), staging diagnosis and follow-up evaluation of cancer patients, we selected the skeletal muscle index (SMI, cm^2^/m^2^) measured by CT within 1 month before surgery to define sarcopenia (4). Concretely, the mean areas of total skeletal muscle complement (cm^2^) at the third lumbar vertebra on two consecutive axial CT images was measured, based on thresholds of −29 to +150 Hounsfield units (HU), by a single trained researcher (JKY) using OsiriX software, version 12.0.4 (http://www.osirix-viewer.com) (6). SMI was ultimately derived by standardizing the skeletal muscle area with height (m^2^). The cutoff values for SMI were set as 43 cm^2^/m^2^ for males with body mass index (BMI) <25kg/m^2^, 53 cm^2^/m^2^ for males with BMI ≥25kg/m^2^, and 41 cm^2^/m^2^ for females (5).

### Markers of systemic inflammation

Plenty of inflammation-based prognostic scores reflecting systemic inflammatory response (SIR) were calculated by laboratory serum parameters within 1 week before surgery (19). The NLR, along with lymphocyte-to-monocyte ratio (LMR), platelet-to-lymphocyte ratio (PLR), systemic immune-inflammation index (SII), prognostic nutritional index (PNI) and mGPS, was selected in this study and reportedly associated with unfavorable prognosis of multiple tumors, including RCC (11, 19, 20). The optimal cutoff values of these indices were calculated by the X-tile software, version 3.6.1 (Yale University, New Haven, Connecticut) (21), a software to provide global assessment of all possible divisions of a population into three or two marker expression levels, to select the optimal division, and to visualize the robustness of the relationship between a biomarker and outcome. The inflammatory markers and their optimal cutoff values were listed in **Supplementary Table S1**, respectively.

### Follow-up investigation

The study was followed until March 2021, and most routine follow-up appointments included a physical examination, clinical laboratory tests, an abdominal ultrasonography, or a chest and abdominal CT examination as required. OS was defined as the time ranging from surgery to death from any cause or the last follow-up. Clinical variables and survival outcomes of patients were collected by two independent authors (X Chen, J Yang).

### Statistical analysis

Firstly, cohort characteristics and systemic inflammatory markers between preoperative sarcopenia or nonsarcopenia groups were analyzed by the Student’s T test or Mann-Whitney U test for continuous variables, and the Chi-squared test or Fisher’s exact test for categorical variables. Based on clinical significance and prior knowledge from previously published literatures, specific covariates associated with survival were selected. Logistic regression analysis was performed to determine the relationship between sarcopenia and inflammatory markers. Then the covariates with p-value<0.05 in univariable Cox regression analysis were chosen for LASSO regression analysis, and the identified significant factors were subsequently included in multivariable Cox regression analysis to extract independent predictors of survival. Kaplan-Meier survival curves were plotted by GraphPad Prism version 8.0.2 and their differences were examined using the log-rank test. Finally, screened independent predictors were incorporated in a nomogram for predicting the probability of 1- and 3-year OS for RCC patients undergoing nephrectomy. The nomogram was internally validated with R version 4.1.3 using 1000-sample bootstrapped validation, a statistical method in which multiple evolutionary trees are constructed to check model confidence by repeatedly sampling data sets. In particular, concordance index (C-index), calculated based on the result of Cox proportional hazards model by “survival” package in R, was used to evaluate the discrimination of the model by estimating the probability of concordance between predicted and observed value ranging from 0.5 to 1.0. Meanwhile, calibration and clinical significance of the model were assessed by 1000-sample bootstrapped calibration plots and Kaplan-Meier curves, in which patients were stratified into high-risk and low-risk group according to their nomogram scores by X-tile software. To compare predictive ability of the nomogram with clinical stage-based, sarcopenia-based and integrated “NLR+PLR+mGPS” methods in predicting 1-, 2-, and 3-year survival in patients with RCC, net reclassification index (NRI), integrated discriminant improvement (IDI) and decision curve analysis (DCA) were utilized. Statistical analyses were carried out using SPSS 19.0 (SPSS Corporation, Armonk, NY, USA) and R version 4.1.3. All tests were two sided, and P <0.05 was considered statistically significant.

## Results

### Demographic features, clinicopathological characteristics, and correlations between sarcopenia and systemic inflammation

With a median follow-up of 20.00 months, the characteristics of total 276 patients classified by preoperative SMI were presented in **Table 1**. 96 patients (34.8%) were classified as sarcopenia, and 25 patients (9.1%) died during the follow-up. The mean age of patients was 57.8 years, and a majority were males (68.8%). Clinical stage classified 62.0%, 4.0%, 29.3% and 4.7% of the cancers as stages I, II, III and IV. Less than 5% (9 in detail) patients in stage M1 underwent cytoreductive surgery to delay disease progression in combination with interferon, sorafenib or sunitinib therapy. Patients with sarcopenia were significant in: older age, high proportion of female, lower BMI and triglyceride, bigger in tumor size, undergoing RN, having stage II or III (vs I) cancer, and shorter survival time. However, no significant differences were observed in hypertension, diabetes, pathologic category, tumor location, uric acid, serum creatinine, urea, or high-density lipoprotein cholesterol between these two groups.

**Table 1:** Baseline characteristics with comparison between sarcopenia and nonsarcopenia patients.

Characteristic	TotalNo.(%)	SMI, cm^2^/m^2^		P value
		SarcopeniaNo.(%)	NonsarcopeniaNo.(%)	
Total patients	276 (100)	96 (34.8)	180 (65.2)	
Age (years)	58.5 (19-87)	64.0 (26-87)	54.5 (19-83)	**<0.001**
**Age categorized (years)**	≤65>65	193 (69.9) 83 (30.1)	57 (59.4) 39 (40.6)	136 (75.6) 44 (24.4)	**0.005**
**Gender** Male Female	190 (68.8) 86 (31.2)	57 (59.4) 39 (40.6)	133 (73.9) 47 (26.1)	**0.013**
BMI (kg/m^2^)	24.39 ± 3.31	23.19 ± 3.11	25.03 ± 3.24	**<0.001**
**BMI categorized (kg/m^2^)** <25 ≥25	158 (57.2) 118 (42.8)	56 (58.3) 40 (41.7)	102 (56.7) 78 (43.3)	0.790
**Hypertension** No Yes	157 (56.9) 119 (43.1)	57 (59.4) 39 (40.6)	100 (55.6) 80 (44.4)	0.542
**Diabetes** No Yes	228 (82.6) 48 (17.4)	74 (77.1) 22 (22.9)	154 (85.6) 26 (14.4)	0.077
**Pathologic categorized** ccRCC pRCC chRCC Others	233 (84.4) 15 (5.4) 13 (4.7) 15 (5.4)	86 (89.6) 3 (3.1) 2 (2.1) 5 (5.2)	147 (81.7) 12 (6.7) 11 (6.1) 10 (5.6)	0.253
**Tumor location** Upper Middle Lower Mixed	62 (22.5) 77 (27.9) 80 (29.0) 57 (20.7)	22 (22.9) 33 (34.4) 26 (27.1) 15 (15.6)	40 (22.2) 44 (24.4) 54 (30.0) 42 (23.3)	0.240
**Tumor size (cm)** ≤4 >4&≤7 >7	123 (44.6) 108 (39.1) 45 (16.3)	31 (32.3) 44 (45.8) 21 (21.9)	92 (51.1) 64 (35.6) 24 (13.3)	**0.009**
**Surgical options** RN PN	143 (51.8) 133 (48.2)	61 (63.5) 35 (36.5)	82 (45.6) 98 (54.4)	**0.004**
**Clinical stage** I II III IV	171 (62.0) 11 (4.0) 81 (29.3) 13 (4.7)	49 (51.0) 7 (7.3) 36 (37.5) 4 (4.2)	122 (67.8) 4 (2.2) 45 (25.0) 9 (5.0)	**0.016**
UA (umol/L)	340.3 (166.3-776.1)	326.1 (180.8-576.8)	353.0 (166.3-776.1)	0.181
Scr (umol/L)	66.4 (38.5-211.2)	65.4 (40.9-121.0)	67.5 (38.5-211.2)	0.363
Urea (mmol/L)	5.4 (3.0-17.7)	5.7 (3.0-10.4)	5.3 (3.1-17.7)	0.771
HDL-C (mmol/L)	1.1 (0.5-2.4)	1.1 (0.6-2.4)	1.1 (0.5-2.3)	0.493
TG (mmol/L)	1.3 (0.3-8.7)	1.2 (0.5-3.2)	1.5 (0.3-8.7)	**0.001**
OS (months)	20.0 (3-39)	16.0 (3-35)	21.0 (7-39)	**<0.001**

SMI, skeletal muscle index; ccRCC, clear cell renal cell carcinoma; pRCC, papillary renal cell carcinoma; chRCC, chromophobe renal cell carcinoma; RN, radical nephrectomy; PN, partial nephrectomy; UA, uric acid; Scr, serum creatinine; HDL-C, high-density lipoprotein cholesterol; TG, triglyceride; OS, overall survival. The values in bold means P < 0.05.

The comparisons of systemic inflammatory markers between sarcopenia and nonsarcopenia groups were shown in **Supplementary Table S2**, where sarcopenia patients tended to have lower albumin, higher NLR and lower PNI (all P<0.05). According to **Table 2**, multivariable logistic regression analysis indicated that only age (P=0.029), gender (P=0.005) and NLR (P=0.004) were independent predictors of sarcopenia after adjusted for the variables of BMI, tumor location and size, clinical stage, and systemic inflammatory markers.

**Table 2:** Logistic regression analysis between clinicopathologic variables and sarcopenia.

Characteristic	Univariable analysis		Multivariable analysis	
	OR (95% CI)	P value	OR (95% CI)	P value
**Age categorized (years)** ≤65 >65	Reference 2.12 (1.24-3.60)	**0.006**	Reference 1.94 (1.07-3.53)	**0.029**
**Gender** Male Female	Reference 1.94 (1.14-3.28)	**0.014**	Reference 2.29 (1.28-4.10)	**0.005**
**BMI categorized (kg/m^2^)** <25 ≥25	Reference 0.93 (0.57-1.54)	0.790		
**Tumor location** Upper Middle Lower Mixed	Reference 1.36 (0.69-2.72) 0.88 (0.44-1.76) 0.65 (0.30-1.43)	0.377 0.709 0.282		
**Tumor size (cm)** ≤4 >4 & ≤7 >7	Reference 2.04 (1.17-3.57) 2.60 (1.27-5.30)	**0.012** **0.009**		0.062 0.239
**Clinical stage** I II III IV	Reference 4.36 (1.22-15.55) 1.99 (1.15-3.45) 1.11 (0.33-3.76)	**0.023** **0.014** 0.871		0.307 0.584 0.517
**NLR** <2.64 ≥2.64	Reference 2.64 (1.58-4.39)	**<0.001**	Reference 2.43 (1.33-4.43)	**0.004**
**LMR** <2.88 ≥2.88	Reference 0.66 (0.37-1.17)	0.157		
**PLR** <151.23 ≥151.23	Reference 1.32 (0.79-2.21)	0.293		
**SII** <482.30 ≥482.30	Reference 1.33 (0.81-2.18)	0.262		
**PNI** <43.50 ≥43.50	Reference 0.42 (0.20-0.89)	**0.023**		0.914
**mGPS** 0 1 2	Reference 0.80 (0.34-1.89) 2.65 (1.07-6.56)	0.603 **0.035**		0.127 0.659

OR; odd ratio, CI; confidence interval, NLR; neutrophil-to-lymphocyte ratio, LMR; lymphocyte-to-monocyte ratio, PLR; platelet-to-lymphocyte ratio, SII; systemic immune-inflammation index, PNI; prognostic nutritional index, mGPS; modified Glasgow Prognostic Score. The values in bold means P < 0.05.

### Survival analysis and kaplan-meier curves of sarcopenia, systemic inflammatory markers, and the combinations

**Figure 2:**
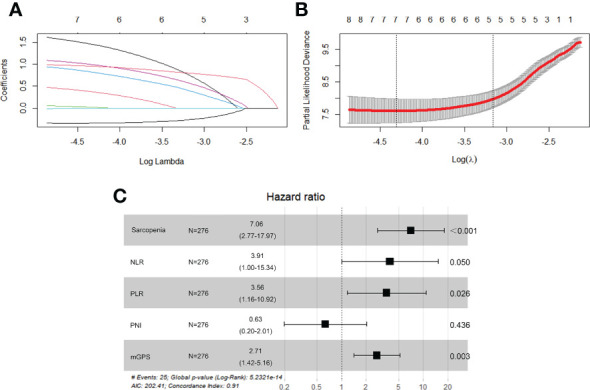
LASSO regression analysis and forest plot of multivariable Cox regression analysis. **(A)** The variation trajectory of each independent variable. The logarithm of the independent variable lambda was taken as the horizontal axis, and the coefficient of the independent variable was taken as the vertical axis. **(B)** Confidence intervals for each phase for each lambda, the vertical black dotted lines defined the optimal values of lambda, which provides the best fit. **(C)** Independent predictors screened by multivariable Cox regression analysis and presented as forest plot. NLR, neutrophil-to-lymphocyte ratio; PLR, platelet-to-lymphocyte ratio; PNI, prognostic nutritional index; mGPS, modified Glasgow Prognostic Score.

3-year OS for the entire cohort was 87.0%, and 3-year cumulative survival rate was 74.9% for sarcopenia group compared with 92.7% for nonsarcopenia group (P<0.001). **Table 3** illustrated variables associated with OS in univariable Cox hazards regression analysis, including age, tumor size, clinical stage, preoperative sarcopenia, NLR, LMR, PLR, SII, PNI and mGPS (all P<0.05). To check for collinearity and to avoid overfitting of the model, a LASSO regression analysis was conducted and five significant predictors (including sarcopenia, NLR, PLR, PNI and mGPS) were screened, as shown in **Figure 2**. In multivariable analyses, except PNI (P=0.436), sarcopenia (HR, 7.06; 95% CI, 2.77-17.97; P<0.001), NLR (HR, 3.91; 95% CI, 1.00-15.34; P=0.050), PLR (HR, 3.56; 95% CI, 1.16-10.92; P=0.026), and mGPS (HR, 2.71; 95% CI, 1.42-5.16; P=0.003) were independent prognostic variables for OS.

**Table 3:** Univariable Cox hazards regression analysis of clinicopathologic variables in relation to OS of RCC patients following nephrectomy.

Characteristic	Univariable analysis	
	HR (95% CI)	P value
**Age categorized (years)** ≤65 >65	Reference 2.99 (1.36-6.59)	**0.007**
**Gender** Male Female	Reference 1.09 (0.47-2.52)	0.850
**BMI categorized (kg/m^2^)** <25 ≥25	Reference 0.50 (0.21-1.20)	0.120
**Tumor location** Upper Middle Lower Mixed	Reference 1.72 (0.59-5.05) 0.88 (0.25-3.03) 1.15 (0.33-3.96)	0.321 0.833 0.831
**Tumor size (cm)** ≤4 >4&≤7 >7	Reference 2.12 (0.71-6.34) 7.52 (2.60-21.73)	0.178 **<0.001**
**Clinical stage** I II III IV	Reference 3.53 (0.76-16.37) 2.63 (1.09-6.34) 8.48 (2.21-32.46)	0.107 **0.032** **0.002**
**Sarcopenia** No Yes	Reference 5.19 (2.23-12.06)	**<0.001**
**NLR** <2.64 ≥2.64	Reference 12.43 (3.72-41.58)	**<0.001**
**LMR** <2.88 ≥2.88	Reference 0.17 (0.08-0.37)	**<0.001**
**PLR** <151.23 ≥151.23	Reference 8.88 (3.33-23.69)	**<0.001**
**SII** <482.30 ≥482.30	Reference 4.10 (1.63-10.27)	**0.003**
**PNI** <43.50 ≥43.50	Reference 0.10 (0.04-0.22)	**<0.001**
**mGPS** 0 1 2	Reference 7.45 (2.87-19.35) 18.55 (6.93-49.69)	**<0.001** **<0.001**

HR; hazard ratio, CI; confidence interval, NLR; neutrophil-to-lymphocyte ratio, LMR; lymphocyte-to-monocyte ratio, PLR; platelet-to-lymphocyte ratio, SII; systemic immune-inflammation index, SII; systemic immune-inflammation index, PNI; prognostic nutritional index, mGPS; modified Glasgow Prognostic Score. The values in bold means P < 0.05.

**Figure 3:**
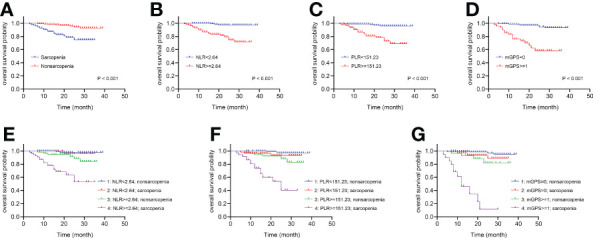
The Kaplan-Meier curves of sarcopenia, systemic inflammatory markers and the combinations for patients following nephrectomy. **(A)** OS for patients with or without sarcopenia. **(B)** OS for patients with high or low NLR. **(C)** OS for patients with high or low PLR. **(D)** OS for patients with high or low mGPS. **(E)** Four combinations of sarcopenia and NLR. **(F)** Four combinations of sarcopenia and PLR. **(G)** Four combinations of sarcopenia and mGPS. OS, overall survival; NLR, neutrophil-to-lymphocyte ratio; PLR, platelet-to-lymphocyte ratio; mGPS, modified Glasgow Prognostic Score.

The Kaplan-Meier curves depicted in **Figures 3A–D** indicated that patients with sarcopenia, NLR ≥2.64, PLR ≥151.23 and mGPS ≥1 (no significance between mGPS of 1 and 2, P =0.112) tended to have worse survival (all log-rank P <0.001). Something interesting happened when sarcopenia was combined respectively with systemic inflammatory markers above. **Figure 3E** presented no significant differences between sarcopenia and nonsarcopenia groups when NLR <2.64 (P =0.720), while there were significant differences between the two groups when NLR ≥2.64 (P <0.001). Moreover, the differences between low and high NLR groups were observed in sarcopenia patients (P <0.001), as well as that in nonsarcopenia patients (P =0.014). In particularly, patients with both sarcopenia and NLR ≥2.64 were estimated with worst survival. The similar pattern could also be found when combining sarcopenia with PLR or mGPS respectively in **Figures 3F, G**.

### Construction and internal validation of the nomogram based on sarcopenia and systemic inflammation

**Figure 4:**
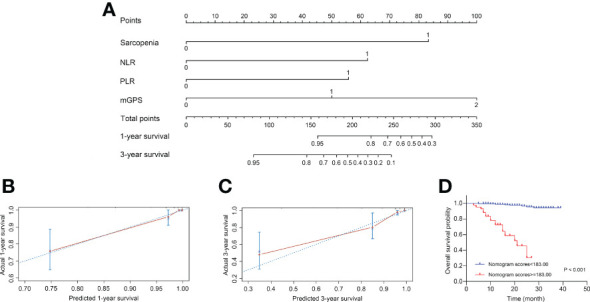
Construction and internal validation of the nomogram for predicting the 1- and 3-year OS of patients with RCC following nephrectomy. **(A)** Nomogram for predicting the 1- and 3-year OS. **(B)** Calibration plot of the nomogram for 1-year survival. **(C)** Calibration plot of the nomogram for 3-year survival. **(D)** The Kaplan-Meier curves for patients with high or low risk stratified by the nomogram scores. OS, overall survival; RCC, renal cell carcinoma; NLR, neutrophil-to-lymphocyte ratio; PLR, platelet-to-lymphocyte ratio; mGPS, modified Glasgow Prognostic Score.

Based on above findings, a novel nomogram integrated four independent predictors, i.e., sarcopenia, NLR, PLR and mGPS, was developed for predicting 1- and 3-year OS of patients with RCC after nephrectomy, as shown in **Figure 4A**. Each variable was assigned a score on the basis of its contributions in the nomogram, and the predicted probability of patients’ survival time could forecast according to the sum of points. The C-index of the model was 0.907 (95% CI, 0.882–0.931), and the calibration plots were well displayed in **Figures 4B, C**. After the optimal cutoff value for scores was calculated from the nomogram using X-tile software (scores <183.00 classified as low-risk group, and scores ≥183.00 classified as high-risk group), the Kaplan-Meier curve plotted in **Figure 4D** revealed the clinical significance of this model (P <0.001). The mean survival time was 37.89 months for low-risk group, compared with 19.05 months for high-risk group. In addition, **Supplementary Figure S1** depicted the predictability of the nomogram in ccRCC subgroup (P <0.001).

### Comparison of the nomogram based on sarcopenia and systemic inflammation with other methods

**Figure 5:**
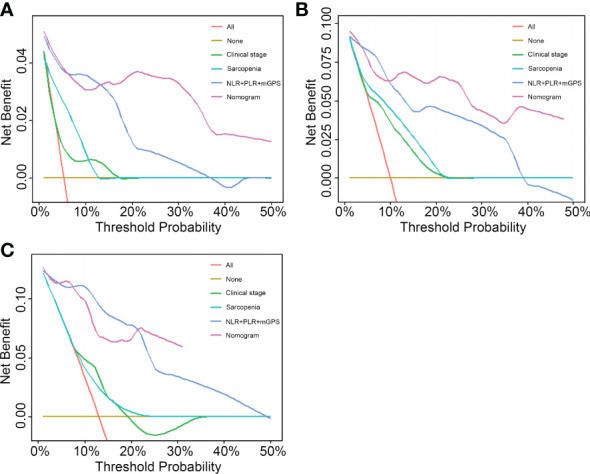
Performance comparison of the nomogram with clinical stage-based, sarcopenia-based and integrated “NLR+PLR+mGPS” methods. **(A)** 1-year decision curve analysis of the four models. **(B)** 2-year decision curve analysis of the four models. **(C)** 3-year decision curve analysis of the four models. NLR, neutrophil-to-lymphocyte ratio; PLR, platelet-to-lymphocyte ratio; mGPS, modified Glasgow Prognostic Score.

To compare the predictive ability of the nomogram with clinical stage-based, sarcopenia-based and integrated “NLR+PLR+mGPS” methods, both NRIs and IDIs shown in **Table 4** were greater than 0. **Figure 5** presented DCAs in predicting 1-, 2-, and 3-year survival, disclosed a net benefit of the nomogram at a threshold probability greater than 20%.

**Table 4:** NRI and IDI used to compare predictive ability of the nomogram with other methods in predicting 1-, 2- and 3-year survival of RCC patients.

	NRIEstimates (95% CI)	IDIEstimates (95% CI)
	**1 year**	
Nomogram vs. Clinical stage Nomogram vs. Sarcopenia Nomogram vs. NLR+PLR+mGPS	0.76 (0.26-1.07) 0.76 (0.36-1.13) 0.37 (-0.13-1.03)	0.29 (0.13-0.52) 0.28 (0.13-0.48) 0.14 (0.03-0.28)
	**2 year**	
Nomogram vs. Clinical stage Nomogram vs. Sarcopenia Nomogram vs. NLR+PLR+mGPS	0.75 (0.30-1.05) 0.79 (0.45-1.23) 0.55 (-0.04-0.86)	0.39 (0.21-0.60) 0.37 (0.26-0.51) 0.19 (0.06-0.33)
	**3 year**	
Nomogram vs. Clinical stage Nomogram vs. Sarcopenia Nomogram vs. NLR+PLR+mGPS	0.70 (0.27-1.14) 0.83 (0.18-1.27) 0.15 (-0.23-0.75)	0.43 (0.24-0.64) 0.34 (0.25-0.48) 0.19 (0.06-0.35)

CI, confidence interval; NRI, net reclassification index; IDI, integrated discrimination improvement; NLR, neutrophil-to-lymphocyte ratio; PLR, platelet-to-lymphocyte ratio; mGPS, modified Glasgow Prognostic Score. The values in bold means P < 0.05.

## Discussion

Although clinical strategies have accumulated, there is still a lack of simple, practicable and widely accessible preoperative prognostication in management of RCC. Taking a wide range of inflammatory variables previously reported that could be linked to RCC prognosis into consideration was one of the strengths of this study. The diagnostic criteria and prevalence of sarcopenia (34.8% of total 276 patients) were similar to the reported studies in RCC (4, 16). What we found between sarcopenia and greater NLR is consistent with prior literature (16), and the effects of targeted therapy and immunotherapy on serum inflammatory indexes may help explain the differences in the correlation between PLR and sarcopenia risk between two studies. Moreover, we newly suggested that preoperative sarcopenia along with elevated systemic inflammatory markers, e.g., NLR, PLR and mGPS, was linked to inferior OS in our crowd, which agrees with previous studies. Higgins et al. (14) found the combined utility of sarcopenia and the elevated mGPS for predicting reduced survival and earlier recurrence in patients with localized RCC. Sarcopenia with elevated NLR or CRP as a negative predictor of OS after cytoreductive nephrectomy in metastatic RCC was later investigated (15). Similarly, the poor prognostic effects of sarcopenia and NLR in colorectal cancer (11), as well as sarcopenia and LMR in gastric cancer (13) have explored.

There are still some findings that differ from previous studies. BMI has previously been regarded as a prognostic indicator of tumors. In this paper, sarcopenia was found associated with a low BMI, while multivariable analysis revealed that BMI was not an independent predictor for OS, which was in agreement with prior studies examining BMI and RCC (15, 22). However, a recent research pointed out that longer survival occurred in patients with higher BMI, and it was restricted to males, but not to females (23). Huszno et al. (24) also discovered that lower BMI was a significant predictor of worse OS in metastatic RCC. Thus, the impact of BMI on prognosis of patients with RCC needs further study, and sarcopenia seems to be a more comprehensive and accurate indicator in body composition reflection than BMI. Furthermore, a possible explanation for the irrelevance of clinical stage to OS is that the number of patients in stage II and IV is too small compared with those in stage I and III, leading to inevitable statistical bias. The staging system could have limited practical value, when almost all patients are divided into one group or subgroup (25), e.g., all patients with T2 tumors were staged in the T2a subgroup, and 92.6% of patients (75/81) with T3 tumors were staged in the T3a subgroup in the present study.

Sarcopenia, acting as an important physiological change in underlying emaciation and weakness caused by tumor progression, is the result of tumor-host interaction. Several metabolic and inflammatory factors and molecular pathways are involved in the onset and progression of sarcopenia, which is classified as primary sarcopenia (just age-related) and secondary sarcopenia (caused by disuse, neurodegenerative disease, inflammatory disease or cachexia) (26). Multiple studies have recently shown that sarcopenia is related to severe postoperative complications (27), inferior survival (4, 22), and dose limiting toxicity (28) in patients with RCC. In the current study, sarcopenia is perceived as a negative prognostic factor in patients.

Besides sarcopenia, SIR also takes a crucial part in tumor cachexia. Increased neutrophils can promote tumor growth and metastasis by remodeling the extracellular matrix, as well as inhibiting the immune system through suppressing the cytolytic activity of immune cells such as lymphocytes (29). In addition, tumor cells are deemed to overcome the damage from immune system and mechanical trauma when covered with platelets and promote its growth *via* the vascular endothelial growth factor release from platelets (30). Conversely, lymphocytes and interferon (IFN)-γ could collaborate to prevent primary tumor development and shape tumor immunogenicity (31). The above may help explain the essential role of up-regulated NLR and PLR in the prognosis of our cohort (due to increased neutrophil and platelet counts, and decreased lymphocyte counts). On the other side, inflammatory cytokines such as interleukin (IL) 6 is thought to increase the synthesis of CRP and decrease the synthesis of albumin in the liver (32), the two elements contained in mGPS. This helps explain the association between higher mGPS and reduced OS in our study. It’s worth noting that the mGPS, compared with NLR and PLR, is superior in differentiating favorable from poor prognostic groups in tumors (20), thus is recommended for routine assessment of patients with cancer.

Not only are sarcopenia and inflammation respectively associated with tumor progression leading poor survival, but tumor-mediated inflammation could in turn exacerbate muscle loss and further create a tumor-pointing vicious cycle between sarcopenia and inflammation. Tumor necrosis factor (TNF)-α, as one of proinflammatory cytokines in tumor cachexia, is responsible for several metabolic derangements and stimulates catabolism of muscle mainly by activation of the ubiquitin-proteasome system (UPS) (33) and nuclear factor of kappa-B (NF-κB) (34) signaling pathway. Both elevated Smad2/3 and NF-κB seems to induce protein degradation separately by the blockade of Akt and the upregulation of muscle ring finger protein 1 (MuRF1) (35). Moreover, increased oxidative stress has significant effects on mitochondrial function, sarcolemmal integrity, and modulation of skeletal muscle during cancer (36). It is worth noting that muscle loss could in turn bring about local inflammation in muscles, leading to further muscle breakdown and inflammation status exacerbation (8). We found that only NLR, compared with other inflammatory markers, was screened as one of independent predictors of sarcopenia. It would be potentially resulted from a vicious cycle existed between muscle damage and neutrophils: muscle damage combined with immune ageing could lead to inefficient neutrophil migration, which was associated with dysregulation of constitutive phosphoinositide 3-kinase (PI3K)-Akt pathway and would in turn cause secondary damage to healthy muscles (37).

These findings should be interpreted with caution, as several limitations exist. Firstly, it is a retrospective, single-center study, leading to inevitable patient selection bias, and multi-center studies will be performed for further external validation of these results. It is also difficult to assess long-term outcomes because of the incomplete medical records and imaging data prior to January 2018. Secondly, the prognostic value of sarcopenia measured by CT images alone at a given point in time is limited to its heterogeneous dynamic process. And the importance of diagnostic criteria of sarcopenia differs in the two European consensuses: muscle strength (38) is recommended as the most important factor in the new version, rather than low muscle mass (26) in the 2010 consensus. Finally, the nomogram is based on the result of an oriental group, and its applicability in western populations should be comprehensively validated.

## Conclusions

In the present study, sarcopenia was associated with systemic inflammation, measured as NLR, in patients following nephrectomy. Four factors, i.e., sarcopenia, NLR, PLR and mGPS, were found as independent predictors of OS and integrated in a novel nomogram for risk stratification, prognosis prediction and personalized therapeutics of patients with RCC. The potential mechanisms of interactions between tumor, sarcopenia and inflammation were then uncovered. More clinical validation using multi-center data will be performed in the next-step work.

## Data availability statement

The original contributions presented in the study are included in the article/**Supplementary Material**. Further inquiries can be directed to the corresponding authors.

## Ethics statement

The studies involving human participants were reviewed and approved by the Ethics Committee of the First Affiliated Hospital of Soochow University with approval number No.(2021)332. Written informed consent for participation was not required for this study in accordance with the national legislation and the institutional requirements.

## Author contributions

QL and JJY designed the study, performed data collection and analysis, and drafted the manuscript. XC, JKY and XZ collected part of the clinical data, helped with patient follow-up and manuscript draft. XZ and YH contributed to acquisition of data and critically appraised the manuscript. JP and YL designed the study, supervised and directed the project. All authors read and approved the final version, and agreed to publish the manuscript.
