# Supplementary material for: Prognostic significance of sarcopenia and systemic inflammation for patients with renal cell carcinoma following nephrectomy
Source: Front Oncol. 2022 Dec 15;12:1047515. doi: 10.3389/fonc.2022.1047515 (PMC9798277; doi:10.3389/fonc.2022.1047515)
Supplement: Supplementary file 1 [file DataSheet_1.doc]

**Supplementary Table S1** Systemic inflammation-based prognostic scores.

| **Systemic inflammatory markers** | **Score**^a^ |
| --- | --- |
| **Neutrophil-to-lymphocyte Ratio (NLR)**  Neutrophil count : lymphocyte count < 2.64:1  Neutrophil count : lymphocyte count ≥ 2.64:1 | 0  1 |
| **Lymphocyte-to-monocyte Ratio (LMR)**  Lymphocyte count : monocyte count < 2.88:1  Lymphocyte count : monocyte count ≥ 2.88:1 | 0  1 |
| **Platelet-to-lymphocyte Ratio (PLR)**  Platelet count : lymphocyte count < 151.23:1  Platelet count : lymphocyte count ≥ 151.23:1 | 0  1 |
| **Systemic Immune-inflammation Index (SII, ×10^9^/l)**  Platelet count × neutrophil count / lymphocyte count < 482.30  Platelet count × neutrophil count / lymphocyte count ≥ 482.30 | 0  1 |
| **Prognostic Nutritional Index (PNI)**  Albumin (g/l) + 5 × lymphocyte count (×10^9/l) < 43.50  Albumin (g/l) + 5 × lymphocyte count (×10^9/l) ≥ 43.50 | 0  1 |
| **The modified Glasgow Prognostic Score (mGPS)**  C-reactive protein ≤ 10 mg/l and albumin ≥ 35 g/l  C-reactive protein ≤ 10 mg/l and albumin < 35 g/l  C-reactive protein > 10 mg/l  C-reactive protein > 10 mg/l and albumin < 35 g/l | 0  0  1  2 |

^a^The cutoff value of NLR, LMR, PLR, SII, and PNI was calculated by X-tile software.

**Supplementary Table S2** Systemic inflammatory markers with comparison between sarcopenia and nonsarcopenia patients.

| **Characteristic** | **Total**  **No.(%)** | **SMI, cm^2^/m^2^** | | **P value** |
| --- | --- | --- | --- | --- |
|  |  | **Sarcopenia**  **No.(%)** | **Nonsarcopenia**  **No.(%)** |  |
| Total patients | 276 | 96 (34.8) | 180 (65.2) |  |
| LY (10*9/L) | 1.7 (0.4-3.9) | 1.7 (0.6-3.7) | 1.7 (0.4-3.9) | 0.134 |
| NEUT (10*9/L) | 3.8 (1.6-17.0) | 3.9 (1.7-17.0) | 3.8 (1.6-11.7) | 0.177 |
| MONO (10*9/L) | 0.4 (0.1-3.0) | 0.4 (0.1-3.0) | 0.4 (0.2-1.1) | 0.442 |
| PLT (10*9/L) | 216.0 (56.0-520.0) | 202.5 (56.0-438.0) | 223.0 (70.0-520.0) | 0.417 |
| Albumin (g/L) | 42.9 (26.6-53.9) | 40.8 (29.3-53.9) | 43.9 (26.6-52.0) | **0.002** |
| **NLR**  <2.64  ≥2.64 | 163 (59.1)  113 (40.9) | 42 (43.8)  54 (56.3) | 121 (67.2)  59 (32.8) | **<0.001** |
| **LMR**  <2.88  ≥2.88 | 64 (23.2)  212 (76.8) | 27 (28.1)  69 (71.9) | 37 (20.6)  143 (79.4) | 0.156 |
| **PLR**  <151.23  ≥151.23 | 181 (65.6)  95 (34.4) | 59 (61.5)  37 (38.5) | 122 (67.8)  58 (32.2) | 0.293 |
| **SII**  <482.30  ≥482.30 | 145 (52.5)  131 (47.5) | 46 (47.9)  50 (52.1) | 99 (55.0)  81 (45.0) | 0.262 |
| **PNI**  <43.50  ≥43.50 | 32 (11.6)  244 (88.4) | 17 (17.7)  79 (82.3) | 15 (8.3)  165 (91.7) | **0.021** |
| **mGPS**  0  1  2 | 227 (82.2)  28 (10.1)  21 (7.6) | 76 (79.2)  8 (8.3)  12 (12.5) | 151 (83.9)  20 (11.1)  9 (5.0) | 0.072 |

LY: lymphocyte; NEUT: neutrophil; MONO: monocyte; PLT: platelet; NLR: neutrophil-to-lymphocyte ratio; LMR: lymphocyte-to-monocyte ratio; PLR: platelet-to-lymphocyte ratio; CRP: C-reactive protein; SII: systemic immune-inflammation index; PNI: prognostic nutritional index; mGPS: modified Glasgow Prognostic Score.

| 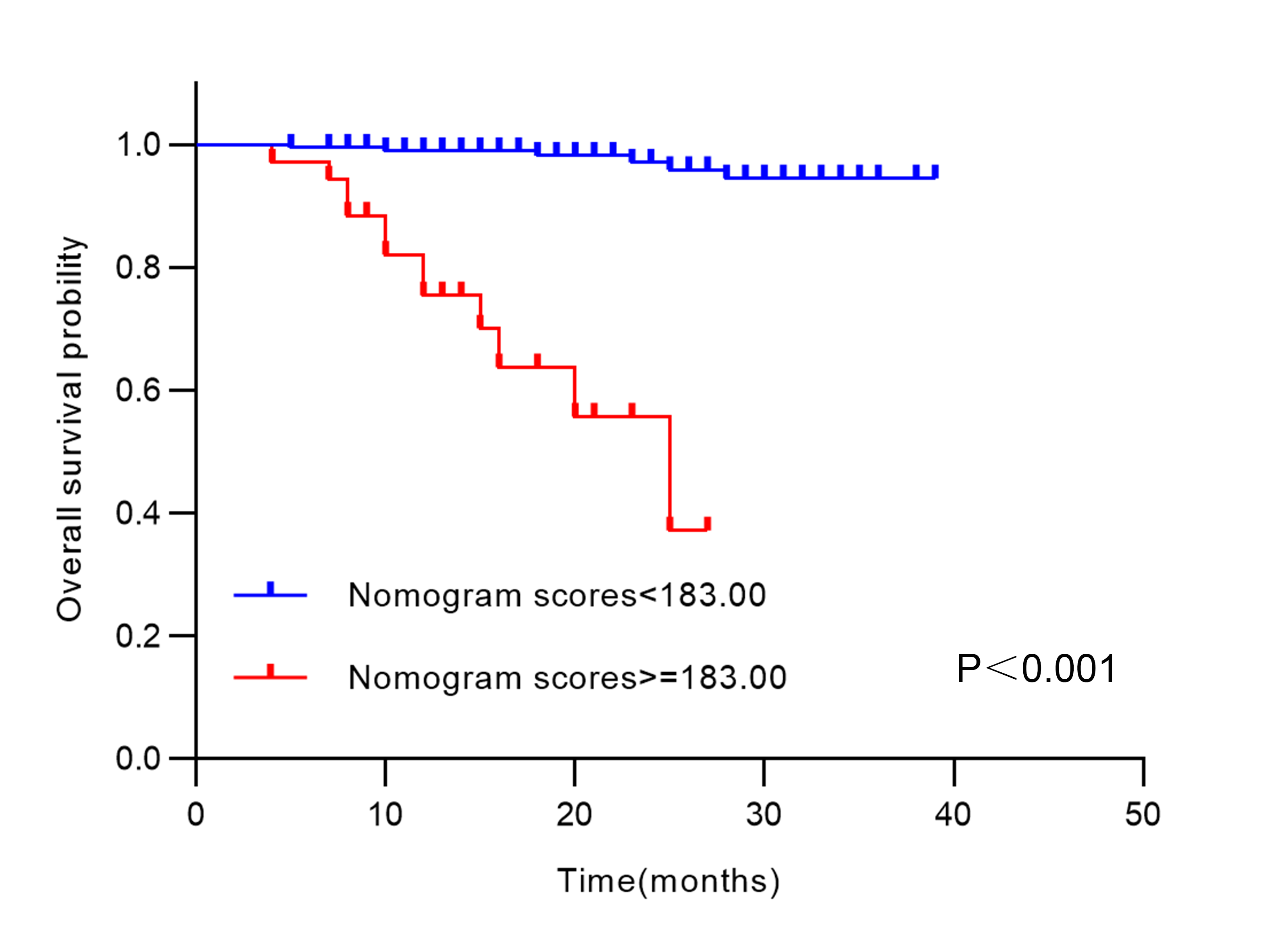 |
| --- |

**Supplementary Figure S1** The Kaplan-Meier curves for clear cell renal cell carcinoma patients with high or low risk stratified by the nomogram scores.
